# Attitudes and Arguments in the Voluntary Assisted Dying Debate in Australia: What Are They and How Have They Evolved Over Time?

**DOI:** 10.3390/ijerph182312327

**Published:** 2021-11-24

**Authors:** Tracee Kresin, Jacinta Hawgood, Diego De Leo, Frank Varghese

**Affiliations:** Australian Institute for Suicide Research and Prevention, WHO Collaborating Centre for Research and Training in Suicide Prevention, School of Applied Psychology, Griffith University, Brisbane, QLD 4122, Australia; tracee.kresin@alumni.griffithuni.edu.au (T.K.); D.Deleo@griffith.edu.au (D.D.L.); ftvarghe@bigpond.net.au (F.V.)

**Keywords:** voluntary assisted dying, euthanasia, assisted suicide, end of life, euthanasia in Australia, right to die, terminally ill, suicide

## Abstract

This paper provides a broad discussion about voluntary assisted dying (VAD) in Australia. The discussion examines the history of the VAD debate in Australia and whether public support for VAD and the arguments that have provided the framework for the VAD debate have evolved over time. This seems a prudent time to have such a discussion, given the very recent rush by all Australian states to bring about or attempt to bring about VAD legislation. This rush, inexplicably perhaps, comes after decades of attempted but failed progress in the legalisation of VAD in Australia. The authors attempted to undertake a systematic literature review for this paper, but the paucity of academic research and the lack of consistent terminology in this area made such a search untenable. Instead, the authors examined parliamentary documentation and then widened the search via the sources found within this documentation. The examination of available data showed that VAD has enjoyed significant public support from Australians over time and that the arguments in the VAD debate in Australia have been consistent over time.

## 1. Introduction

Australia entered into world history in 1995, when the Northern Territory Parliament passed the *Rights of the Terminally Ill Act 1995* (hereafter called ROTTIA), legalising voluntary assisted dying (VAD) in Australia [1]. Just months before this, on 25 March 1995, seven doctors in the state of Victoria went public, announcing that they had all been engaging in VAD—illegally, as there was no VAD legislation in Victoria at that time—and called for a bill to be introduced into Victorian Parliament legalising VAD [2]. These doctors were publicly pilloried and threatened with prosecution, and the call for an inquiry into VAD was summarily rejected. This position became official sometime later, when the then Victorian Premier made an announcement that VAD would not be considered in Victorian Parliament any time in the future, as viewed by the Premier [2].

This was not the end nor the beginning of the debate surrounding VAD in Australia. These were just a few moments in a long history of debate, one that reared into the public arena with painful regularity across the ensuing decades. It was a debate that aroused the vocal passion in many groups, both those that opposed VAD and eventually those that advocated for VAD. It was a debate that was simultaneously played out in the public, with market researchers holding periodic polls on the issue, and in the various Australian parliaments via the submission of more than 58 bills attempting to address the issue of VAD across the decades. 

While these debates, both public and parliamentary, were occurring simultaneously in time, they stayed separate, unaffected and untouched by one another. Public opinion appeared to have no influence over the steps that politicians were prepared to take to legalise VAD in Australia. Similarly, the persistent rejection of attempts to bring the issue of VAD successfully through parliament did not dampen the public support for VAD. Only twice in history, until very recently, did these separate debates come to a common endpoint: the first time in 1995, with the ROTTIA in the Northern Territory, and the next time in 2017, with the passing of the Victorian VAD legislation. This was a remarkable set of circumstances, considering that a large majority of Australians have been in support of VAD since the 1970s [3]. Healey [4] pondered the question as to why politicians did not see the issue of VAD as a great political opportunity, given the high levels of public support over the past few decades. McGee and colleagues [5] asserted that because politicians had appointed themselves as the moral gatekeepers of VAD legislation, it would need a change in the political climate in Australia to bring about legalisation of VAD. 

Overall, since 1978, the majority of the Australian public has supported VAD across the decades. The arguments for and against legalising VAD have remained essentially similar across time. With few exceptions, most analyses that the authors could find showed the same arguments arising each time the issue of VAD rose back into the public arena. There were some arguments that appeared only briefly, but the staple arguments of the 1990s Australian VAD debate remain the staple arguments in the contemporary Australian VAD debate.

This paper aims to provide a broad discussion about the VAD situation in Australia and show how we arrived at where we are now in 2021. The discussion will look at the history of the VAD debate in Australia, the trends in public attitudes toward VAD in Australia, and the trends of the for and against arguments in the VAD debate. The impetus for this discussion came from the very recent and sudden uptake of VAD legislation across the states of Australia in such a short span of time after decades of attempted but failed progress. 

## 2. Terminology

No discourse on VAD can begin without a look at the terminology most commonly used in this field. For the purposes of this paper, VAD refers to “the legal administration of a lethal drug to a terminally ill person at the request of that person” [6] (p.105). VAD is a relatively new phrase in this field in Australia. In the 1990s in Australia, as the debate around VAD came into the spotlight, the term Active Voluntary Euthanasia (AVE) was adopted by many [2] in an effort to differentiate what is now known as VAD from other forms of euthanasia. AVE can be defined as the intentional taking of a patient’s life in response to that patient’s request [7]. 

The term “active” was used to differentiate the act under debate from “passive” euthanasia. The term “active” was used to emphasise that the act was a deliberate act to end the life of a patient [7]. The term “passive” was used to describe those forms of euthanasia involving the withdrawing or withholding of treatment and/or nutrition [7]. The term “voluntary” was used to differentiate the concept under debate from other forms of euthanasia, including non-voluntary euthanasia and involuntary euthanasia [8]. Sikora and Lewins [9] define non-voluntary euthanasia as ending the life of a patient who does not have the capacity to provide consent (e.g., a new-born) and involuntary euthanasia as the ending of the life of a patient either against their will or without their knowledge. As the debate escalated, it was important to define and clarify just exactly what circumstances were being debated.

During the same period of time, the phrase that was in most common use in the United States of America was “physician-assisted suicide” [2]. This term refers to circumstances involving assisted suicide—a mentally competent patient being provided with the means to end their life—where a physician or doctor is the person providing the means [3]. Over the decades since the 1990s, Australia has cycled through a few different terms, including physician-assisted suicide (PAS) and physician-assisted dying (PAD) [10]. Goldney [11] suggested that Australia has moved away from the use of the term PAS because the word “suicide” carries so much stigma with it and that the use of such a word in this context muddies the water for those working in suicide prevention, for the people and the healthcare professionals involved in the act of VAD, and for the authorities that regulate VAD. The adoption of “assisted dying” has given us a term that is more neutral in its connotations. 

VAD is now the term being adopted in the legislation that is emerging throughout Australian jurisdictions and, with media exposure, will likely be the term to be taken up by the public in time. For the sake of consistency and clarification, and because it is the legal term being adopted in Australia, the authors will use the phrase VAD in all discussion regardless of the original term used in the sources being examined. 

## 3. Method

The original intention of the authors was to undertake a systematic literature search into the attitudes and arguments surrounding VAD in Australia over history. Such a search strategy proved unproductive. This was due to both the lack of empirical research into the Australian attitudes about VAD and the paucity of academic research into the arguments comprising the VAD debate in Australia. Instead, the authors turned to parliamentary records, such as inquiries, bills, and committee reports, for example, and records kept by social research groups to support this important discussion. A hand search was then done to identify further literature. The research papers used in the preparation of this discussion were endorsed by all authors.

## 4. History of the VAD Debate in Australia

On 25 May 1995, the Northern Territory in Australia made history by becoming the first jurisdiction in the world to legalise VAD [1]. The ROTTIA became law on 1 July 1996. This new law was promptly challenged in the Northern Territory Supreme Court (on 24 July 1996), where it was ruled as being valid legislation [1]. Not happy with this Supreme Court outcome, a Victorian Member of Parliament (MP), Kevin Andrews, introduced the *Euthanasia Laws Bill 1996* into Federal Parliament in September 1996 [1]. The primary aim of this bill was to bring about new federal legislation that would overrule the ROTTIA, effectively ending VAD in Australia [1]. In Australia, the Federal Parliament can overrule legislation made in its territories but not legislation made in its states [1].

The committee that was charged with considering this 1996 bill received over 12,000 submissions, of which 90% were in opposition to legalising VAD [12]. As noted in later sections, this was in stark contrast to the opinions about VAD held by the Australian public during the same time period. Ultimately, this bill was successful and came into effect on 25 March 1997, effectively outlawing VAD under federal law. The ROTTIA had been in place just nine months when it was overturned [1]. During that period of legalisation, seven people in the Northern Territory made use of the Act [1]. 

Despite the historical significance of these facts, the events of 1995 were not the beginning of the VAD debate in Australia. The Victorian Parliament was the first to raise the issue of VAD in 1985 [13], when it appointed a parliamentary committee with the task of looking into the “right to die” issue [12]. The inquiry by the parliamentary committee generated more than 1000 submissions, included public hearings, and took statements from 152 witnesses [12]. Again, despite the support displayed for VAD by the Australian public at the time, the committee rejected the idea of introducing VAD legislation [13]. 

This rejection set a long-held pattern in Australian parliaments and was just one of many failures to bring about legislation regarding the legalisation of VAD in Australia. Between June 1993, when the very first VAD bill was introduced into an Australian parliament [3], and December 2017, 58 VAD bills went before various Australian parliaments [5]. Apart from the ROTTIA in 1995, all attempts failed [3] until the Victorian Parliament successfully passed the *Voluntary Assisted Dying Act 2017 (Vic)*. Amongst the failures within the various Australian parliaments were bills (draft legislation) that were either rejected or lapsed without action, multiple attempts to repeal the 1997 federal law that prevented the Australian territories from making VAD legislation, and several attempts to bring about a national referendum on the issue of VAD [3].

## 5. Attitudes of Australians toward VAD

In deep contrast to the situation being played out across more than three decades in the various Australian parliaments, the Australian public has long supported the legalisation of VAD [3,12]. Strangely enough though, the mapping of the sociological trends in this public support for VAD has been left largely to public opinion polling organisations. The small amount of research that examined Australian attitudes towards VAD considered almost exclusively the attitudes of healthcare professionals, not the general public [14,15,16]. 

**Figure 1:**
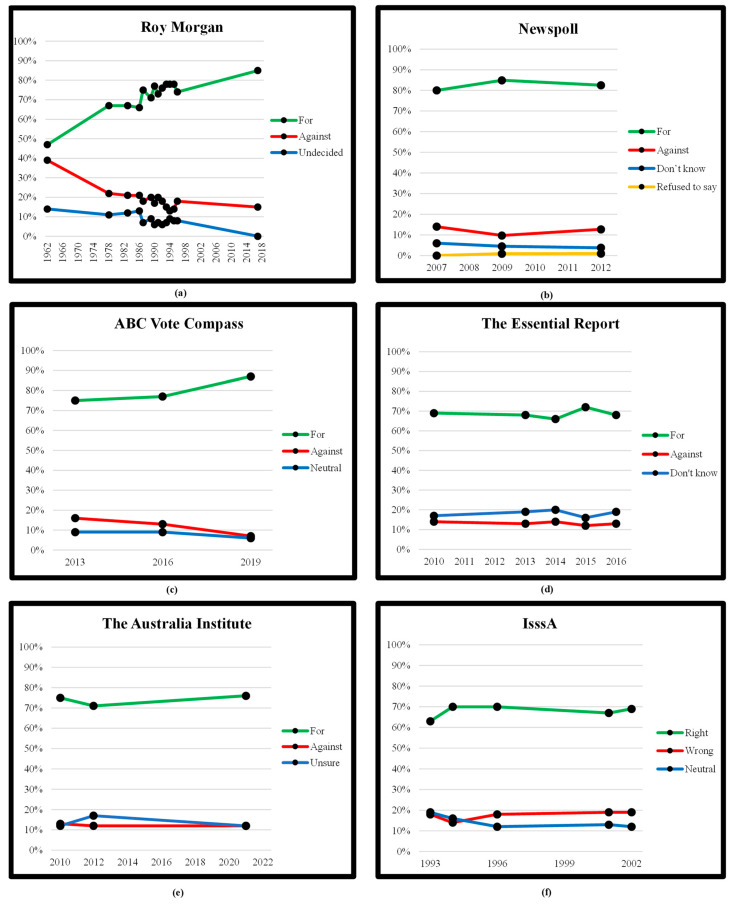
Trends in attitude across time. (**a**) Roy Morgan (**b**) Newspoll (**c**) ABC Vote Compass (**d**) The Essential Report (**e**) The Australia Institute (**f**) IsssA.

In order to track the Australian public’s attitudes toward VAD to examine for any change of attitude over time, we examined the polls undertaken by a number of well-known public opinion polling organisations at various intervals across the past 60-odd years. See Table 1 for the results of these investigations. As can be seen in Table 1, apart from the first public poll undertaken by Roy Morgan [17], the majority of Australians have expressed support for VAD across the years and across the polling organisations. These data were then plotted onto graphs (see Figure 1), which show the overall trends in attitude recorded by each market research group across the years that polls were undertaken by each organisation. The longest running poll, organised by Roy Morgan (Figure 1a) and occurring in various years spanning from 1962 to 2017, shows a clear upward trend in the support for VAD, with steady downward trends in both opposition to VAD and in those remaining uncertain about their position about VAD.

The remaining polls regarding attitudes toward VAD (Figure 1b–f) also show mostly upward trends in the public support for VAD and stable to downwardly moving trends in the opposition to VAD and the unsure/neutral position regarding VAD. It must be noted, however, that these remaining polls cover much shorter periods of time and have much fewer data collection points than the Roy Morgan polls in Figure 1a. They display public opinion across only the past 10–20 years in comparison to the Roy Morgan polls, which cover a period of nearly 60 years. They will then have failed to capture earlier changes in public attitude toward VAD. Certainly, these remaining five polls appear to indicate that public attitudes toward VAD in Australia have been relatively stable across the past 10–20 years.

Surprisingly though, apart from the ROTTIA in 1995, and the more recent success of the Victorian VAD legislation in 2017 (and the subsequent successes and progress in all other Australian states), this overwhelming public support of VAD was not translated into the legalisation of VAD via parliament. The various parliaments of Australia have steadfastly refused to acknowledge the high levels of support for VAD from average Australians. Why it has taken more than 20 years since the introduction of ROTTIA, and subsequent overturning, until 2017 and the passing of the Victorian VAD legislation to effect change in Australia is a question that needs some answers.

## 6. Australian Arguments for and against VAD

While the public opinion polls of the Australian public give an indication of overall attitudes towards VAD across the decades, we examined parliamentary reports, parliamentary inquiries, submissions to parliamentary inquiries, and analyses of the same in an attempt to gather evidence of the prominent arguments for and against VAD. See Table 2 for the results of this investigation.

It can be observed in Table 2, that while some arguments appear briefly in the debate about VAD in Australia and then disappear again, there is a definite core of arguments both for and against that appear across time and across sources.

In a global context, Hendry and colleagues [28] undertook a systematic review of worldwide research that investigated the for and against arguments in the VAD debate across the globe. The papers reviewed by them examined the positions of non-health professionals. Hendry and colleagues concluded that the main arguments on each side of the VAD debate are as follows in Table 3. As can be seen upon examination of Table 2 and Table 3, in essence, the arguments “for” VAD in Australia are similar to those found globally.

In 2020, Walker and colleagues [29] published a study based on the findings of a citizen’s jury in New Zealand. Members of the citizen’s jury were questioned about their attitudes toward VAD and their reasons for holding those attitudes. The main arguments “for” VAD identified in this New Zealand study mirrored those identified in Hendry and colleagues’ [28] paper.

The autonomy/right to die argument features prominently in the Australian VAD debate, as does the argument pushing for compassion and the relief of suffering in those who are terminally ill. Interestingly, the argument in the Australian VAD debate that suggests that VAD should be legalized because it is already occurring here, and it is better that it be done under a regulatory body does not seem to appear in the global arguments for VAD.

Two arguments in support of VAD that are found in Australia and in the New Zealand study—that of the argument that palliative care does not work for everyone and the argument that notes a personal experience of witnessing the death of a loved one as a reason for supporting VAD—seem to have emerged only in more recent times. They appear in the literature from about 2013 forward. These arguments may well be the results of the changes in society that Kerridge and Mitchell [8] and Magnusson [2] suggested may be influencing our attitudes toward and support of VAD in Australia. In particular, advances in healthcare and medical technology, which enables keeping people alive longer [30,31], and a growing population of older adults [32] may have contributed to the emergence of these arguments.

Additionally of note, the two arguments advocating for VAD in Australia that include a mention of suicide do not seem to appear in the global arguments for VAD put forward by Hendry and colleagues [28] nor the Walker and colleagues’ study [29]. The argument that suggests VAD is a safe medical procedure as opposed to the uncertainty and often violence [33] involved in a bid to die by suicide has been showing up in Australian VAD arguments since the mid 1990s. The argument suggesting that legalizing VAD will in fact prevent suicides is a more recent development and may have emerged in line with Australia’s renewed interest and efforts in suicide prevention. While this argument may seem paradoxical, there are some that believe access to VAD is an effective form of suicide prevention. This is based on the notion that offering a terminally ill person the certainty of a way out, even after they have become physically unable to bring about a death by suicide themselves, will in fact give those same people the courage to live longer and past the point of physical incapacitation [34].

In contrast to the few main opposing arguments identified by Hendry and colleagues [28] as being of concern globally-coercion of vulnerable people, physician error in diagnosis, religious reasons, and VAD being morally wrong—both Australia and New Zealand—appear to have established and published a much more comprehensive and extensive range of arguments against VAD.

Of note, the global analysis of arguments against VAD by Hendry and colleagues [28] is devoid of any mention of the concerns about VAD undermining the principles of medicine, VAD negatively impacting the doctor/ patient relationship, and palliative care being sufficient for the purpose. It is also of note that the global analysis specifically excluded the views of medical professionals, while Australian research has focused more heavily on the views and arguments of medical professionals [35,36,37].

The “slippery slope” argument against VAD is mentioned consistently and prominently in every Australian document examined by the authors. This argument essentially suggests that we should not allow a particular practice—in this case, VAD—because allowing it may lead us to eventually allow another practice that is clearly objectionable [38]. In the case of VAD, it is suggested that allowing VAD in its current form will lead to an eventual acceptance and legalization of such a practice in people who are not competent or who are no longer socially valuable [7], children, and those with psychological illness. Yet, this argument did not appear in the global analysis. The arguments that VAD may validate the act of suicide or become a socially accepted and readily available form of suicide have only arisen briefly, to date, in the Australian debate.

## 7. Discussion

In the mid to late 1990s, several authors [2,8] put forward suggestions in an attempt to answer the question, “Why now?” Why was the VAD debate becoming so prominent in Australia in the mid 1990s? What had changed in our society in order to bring this important issue to the surface at that particular point in time? How did the ROTTIA get through parliament and become legislation? Those authors have suggested that we, as a society, had changed and that the attention given to VAD reflected these changes. Kerridge and Mitchell [8] and Magnusson [2] suggested that changes, such as our shifting attitudes toward death, limited health budgets, a decline in the influence of the church on Australian society, advances in healthcare and medical technology, a growing population of older adults, an increase in emphasis on the individual, and the loss of absolute trust in doctors may all have contributed to the circumstances of the 1990s: the perfect environment in which a movement toward VAD could progress.

However, despite the social changes outlined above, as a society, we still somehow failed to transform that seemingly overwhelming public support of VAD into state or federal legislation. It took a further two decades, from the ROTTIA in 1995 and the subsequent overturning of that legislation, to produce VAD legislation once again. Now, after decades of failures, within a matter of four years, Australia has suddenly moved from having no legalisation of VAD to every Australian state now either having legislation in place, legislation passed and waiting to become active, or legislation currently moving through parliament.

We now have a new “Why now?” to be answered. This paper has looked at the status of public attitude toward VAD in Australia from the 1960s onward. This examination has shown a clear early upward trend in support for VAD amongst Australians, with that support held in a stable position across the past 10–20 years. In the same way, this paper has looked at the arguments for and against VAD from about 1995 to now. These appear to be essentially stable over time. So, why now? Why, in 2021, are all Australian states involved in some way in VAD legislation? McGee and colleagues [5] offered up a potential answer to this question. Despite strong public support for VAD consistently since the late 1970s and stable arguments for and against VAD in that same period of time, McGee and colleagues [5] believed that the primary obstacle to legalising VAD in Australia has been a steadfast political unwillingness to consider law reform in this area. However, what has happened to change that previously impenetrable parliamentary opposition? Why now?

Certainly, we should consider Duckett’s [12] view that, while in the early days of the VAD debate, those who opposed VAD were very well organised and very vocal, now—finally—the proponents of VAD have also become better organised and more vocal. Is this the whole story? Was this enough to sway our politicians? The opinion of the Australian public has never before influenced the VAD debate. White and Willmott [38] suggested that there is a combination of contemporary factors that will lead to greater progress in the Australian VAD debate: an international trend towards VAD, growing political support for VAD in Australia, and the weakening of some of the arguments against VAD as empirical evidence is emerging from nations who have already legalised VAD.

Significantly, the authors had to rely upon market research polls to obtain a picture of the public attitudes toward VAD across time in Australia. Even the latest parliamentary committee report published by the Queensland Parliament in 2020 relied upon this market research data for indicators of public attitude toward VAD. This appears to be a major oversight in the history of the VAD debate in Australia. How can such an important social/medical/legal/moral issue have failed to attract the attention of public health or even health law researchers?

## 8. Conclusions

In an article written in 1997, Magnusson [2] suggested that we, as a society, were moving steadily toward the inevitable acceptance of VAD. He also predicted that within one generation, Australians would look upon their views of the 1990s—those views, of course, being the ones that denied terminally ill people the right to receive medical assistance in dying—to be primitive ones [2]. What Magnusson could not have known, though, was that it was not going to be the Australian public that was the greatest obstacle to legalising VAD across the decades but instead our politicians. In the same way, McGee and colleagues [5] and White and Willmott [39] correctly predicted that the passing of the VAD legislation in Victoria would lead to the renewed efforts of other Australian states to introduce VAD legislation.

This investigation was unable to detect any significant evolution in the public attitudes toward VAD since the late 1970s nor any tangible shift in the arguments for and against VAD. These constructs have been reasonably stable across time. This suggests that neither of these factors, or indeed changes in these factors, are likely to be responsible for the sudden emergence of various VAD legislation across Australian states. More research is needed in this area; in particular, the views of average Australian citizens toward VAD need to be properly investigated through structured research, not public opinion polls. This recommendation for empirical research is supported by a study in which Grove and colleagues [40] concluded that the wording used in public opinion poll questions regarding VAD—in particular, highly emotive language—can affect the answers given by participants. VAD is an important societal issue, and it is surprising that Australia has gone so far forward in such a short span of time with little or no academic research into the views held by Australians.

## Figures and Tables

**Table 1:** Public attitudes toward VAD in Australia.

Poll/Year	1962	1978	1983	1986	1987	1989	1990	1991	1992	1993	1994	1995	1996	2001	2002	2007	2009	2010	2012	2013	2014	2015	2016	2017	2019	2021
Roy Morgan [17]																										
For	47%	67%	67%	66%	75%	71%	77%	73%	76%	78%	78%	78%	74%											85%		
Against	39%	22%	21%	21%	18%	20%	17%	20%	18%	15%	13%	14%	18%											15%		
Undecided	14%	11%	12%	13%	7%	9%	6%	7%	6%	7%	9%	8%	8%											0%		
The Australia Institute [18]																										
For																		75%	71%							76%
Against																		13%	12%							12%
Unsure																		12%	17%							12%
Newspoll [19,20,21]																										
For																80%	84.9%		82.5%							
Against																14%	9.7%		12.7%							
Don’t know																6%	4.5%		3.8%							
Refused to say																	0.9%		1%							
ABC Vote Compass [22]																										
For																				75%			77%		87%	
Against																				16%			13%		7%	
Neutral																				9%			9%		6%	
The Essential Report [23]																										
For																		69%		68%	66%	72%	68%			
Against																		14%		13%	14%	12%	13%			
Don’t know																		17%		19%	20%	16%	19%			
IsssA [9]																										
Right										63%	70%		70%	67%	69%											
Wrong										18%	14%		18%	19%	19%											
Neutral/undecided										19%	16%		12%	13%	12%											

**Table 2:** For and against arguments across time in Australia.

Argument/Year	1995 [24]	1996 [12]	1996 [8]	1996 [25]	2010 [7]	2013 [4]	2014 [26]	2015 [12]	2018 [27]
**For**									
Autonomy/right to die	X	Unavailable	X	X	X	X	X	X	X
Relieve suffering/compassion	X	Unavailable			X	X		X	X
Safe medical practice—as opposed to suicide		Unavailable	X		X				
Personal experience of witnessing death		Unavailable					X		X
Already occurring/better to legalise and regulate	X	Unavailable	X	X	X		X		
Palliative care doesn’t work for everyone		Unavailable				X	X	X	
Prevents suicides								X	
**Against**									
Slippery slope	X	X	X	X	X	X	X	X	X
Sanctity of life	X	X	X	X	X	X		X	X
Out of line with indigenous culture		X							
Coercion of vulnerable people	X		X	X		X		X	X
Religious reasons	X	X		X	X	X	X		
Might become accepted, available & efficient alternative to suicide			X						
Undermines the principles of medicine	X	X	X	X	X	X	X		X
Palliative care is enough	X	X	X			X	X	X	X
Possibility of diagnostic error		X	X						
State-sanctioned killing	X				X	X			X
Will impact the Dr/Pt relationship			X	X	X		X	X	X
Morally wrong		X	X	X			X		X
Discourage search for cures					X				
Law lacks the nuances to deal with dying/safeguards won’t work			X					X	
Validates suicide									X

X = argument mentioned.

**Table 3:** Global arguments in the VAD debate.

For	Against
Right to choose	Abuse/coercion of vulnerable people
Desire for autonomy	Physician error (diagnostic)
Control of own life	Religious objections
To relieve pain and suffering	Morally wrong
To die with dignity	
Personal experience of death of a loved one	
